# Differentiation in the genetic basis of stem trichome development between cultivated tetraploid cotton species

**DOI:** 10.1186/s12870-021-02871-4

**Published:** 2021-02-25

**Authors:** Rong Yuan, Yuefen Cao, Tengyu Li, Feng Yang, Li Yu, Yuan Qin, Xiongming Du, Fang Liu, Mingquan Ding, Yurong Jiang, Hua Zhang, Andrew H. Paterson, Junkang Rong

**Affiliations:** 1 The Key Laboratory for Quality Improvement of Agricultural Products of Zhejiang Province, College of Agriculture and Food Science, Zhejiang A&F University, Lin’an, Zhejiang, 311300 Hangzhou China; 2 Institute of Cotton Research, Chinese Academy of Agricultural Science (ICR, CAAS)/State Key Laboratory of Cotton Biology, Anyang, 455000 China; 3 Plant Genome Mapping Laboratory, University of Georgia, Athens, GA 30605 USA

**Keywords:** Cotton, Stem trichome, Seed fiber, Genetic mapping, Correlation analysis, Parallel evolution

## Abstract

**Background:**

Cotton stem trichomes and seed fibers are each single celled structures formed by protrusions of epidermal cells, and were found sharing the overlapping molecular mechanism. Compared with fibers, cotton stem trichomes are more easily observed, but the molecular mechanisms underlying their development are still poorly understood.

**Results:**

In this study, *Gossypium hirsutum* (*Gh*) and *G. barbadense* (*Gb)* were found to differ greatly in percentages of varieties/accessions with glabrous stems and in trichome density, length, and number per trichopore. *Gh* varieties normally had long singular and clustered trichomes, while *Gb* varieties had short clustered trichomes. Genetic mapping using five F_2_ populations from crosses between glabrous varieties and those with different types of stem trichomes revealed that much variation among stem trichome phenotypes could be accounted for by different combinations of genes/alleles on Chr. 06 and Chr. 24. The twenty- six F_1_ generations from crosses between varieties with different types of trichomes had varied phenotypes, further suggesting that the trichomes of tetraploid cotton were controlled by different genes/alleles. Compared to modern varieties, a greater proportion of *Gh* wild accessions were glabrous or had shorter and denser trichomes; whereas a smaller proportion of *Gb* primitive accessions had glabrous stems. A close correlation between fuzz fiber number and stem trichome density was observed in both *Gh* and *Gb* primitive accessions and modern varieties.

**Conclusion:**

Based on these findings, we hypothesize that stem trichomes evolved in parallel with seed fibers during the domestication of cultivated tetraploid cotton. In addition, the current results illustrated that stem trichome can be used as a morphological index of fiber quality in cotton conventional breeding.

**Supplementary Information:**

The online version contains supplementary material available at 10.1186/s12870-021-02871-4.

## Background

Plant trichomes are epidermal cell outgrowths that perform a variety of important functions. There are many types of trichomes, which are characterized by their morphology, location, and nature. Trichomes can be unicellular or multicellular, glandular or non-glandular, and branched or unbranched. Moreover, branched hairs can be dendritic, tufted, or stellate. Plants with trichomes tend to be more resistant to abiotic stresses, such as frost, heat or drought. Although the functions of trichomes are not clear, they are generally believed to provide physical protection to terrestrial plants against insects, pathogens, and herbivores [1].

Their easy manipulation and observation made plant trichomes ideal for studying cell differentiation-related pathways [2, 3]. The trichomes of *Arabidopsis thaliana* have a specialized unicellular structure and are 200–500 μm in length. In *Arabidopsis*, trichome development shares common mechanisms with root epidermal development, each involving closely-related cell fate transcription factors and a similar lateral inhibitive signaling pathway [4]. The process of trichome initiation includes three physiological stages: endore duplication, rapid growth, and branch formation. The regulatory genes and functions of encoded proteins were investigated to determine mechanisms controlling *Arabidopsis* trichome initiation and the early stages of morphogenesis [5, 6]. The key initiation genes encode a MYB, a bHLH, and a WD-40 repeat-containing protein, forming a MYB–bHLH–WD40 complex that effects trichome initiation as a dual regulator [7], while CAPRICE and TRIPTYCHON negatively regulate trichome initiation [8]. Tobacco (*Nicotiana tabacum* L.) leaf epidermal trichomes differ from those of *Arabidopsis* in that they are multicellular. The mechanisms of tobacco trichome formation have both similarities and differences from those of *Arabidopsis* unicellular trichomes, both being controlled by the MYB–bHLH–WD40 regulatory mechanisms, but differing in the functions of the *MYB* genes [9].

Cotton fiber is the most important raw material in the textile industry, and cotton seeds contain high oil and protein contents that are consumed by animals and humans [10]. Cotton fibers are single-celled structures originating from the ovular epidermis, while trichomes originate from aerial epidermis [2, 11], each by similar developmental processes [12]. Trichomes are broadly classified into glandular and non-glandular forms, and their morphology has been described in detail by Turley et al. (2012).

Classical genetic studies revealed that cotton trichome growth is affected by many loci, which were classified into groups*t*_*1*_ to *t*_*5*_ [13, 14]. Wright et al. (1999) mapped five quantitative trait loci (QTLs) for leaf and stem trichomes using an F_2_ population derived from a cross between *Gossypium hirsutum* (*Gh*) and *G. barbadense* (*Gb*) varieties, including a leaf pubescence QTL on Chr. 06 that corresponds to the *t*_*1*_ locus (Kloth 1993), three other leaf pubescence QTLs on Chrs. 01, 07, and 25, and a QTL on Chr. 23 that is associated with stem trichome density. The latter four QTLs were hypothesized to correspond to the *t*_*2*_ to *t*_*5*_ loci [15]. Lacape and Nguyen (2005) also confirmed the location of locus *t*_*1*_, which is a major determinant of leaf pubescence, in the central region of Chr. 06, and additional genes located on seven other chromosomal locations either positively or negatively affect trichome density. In addition, QTLs affecting lint percentage, fiber length, fiber length uniformity and fiber strength were identified in the *t*_*1*_ locus region of Chr. 06, suggesting that this locus also affects cotton fiber quality [16]. In *G. arboreum*, the glabrous mutation was mapped to linkage group A3, which appears to be orthologous to the *t*_*1*_ locus of allotetraploids [17]. A fiber mutation locus, *sma-4* (*ha*), was detected in the same F_2_ population that co-segregated with the glabrous stem, which suggests either a close linkage or common genetic control between lint fibers and leaf/stem trichomes [17, 18].

*Gh* and *Gb*, the two major species used in cotton production, originated from natural hybridization between species resembling *G. raimondii*, the diploid D genome donor, and *G. arboreum*, the diploid A genome donor, an estimated 1–1.5 million years ago [19]. These cultivated tetraploid species display considerable differences in stem trichome morphology, which suggests that they experienced different evolutionary processes, as also indicated by changes in chromosome structure and gene content revealed by re-sequencing of their whole genomes [20]. Although several studies have reported QTL mapping of cotton trichomes and cloning of a few critical genes [15, 16, 21, 22], the mechanisms governing trichome initiation at the molecular level are still unclear. In this paper, we report the classification of stem trichomes, molecular mapping of their underlying genes, and association analysis between the amount of fuzz fiber and stem trichome density. We also discuss possible evolutionary processes that may have influenced the genetic basis of trichome growth during speciation and domestication of tetraploid cotton.

## Results

### Inter-specific and intra-specific variations in the stem trichome phenotypes of two cultivated tetraploid cottons

**Fig. 1:**
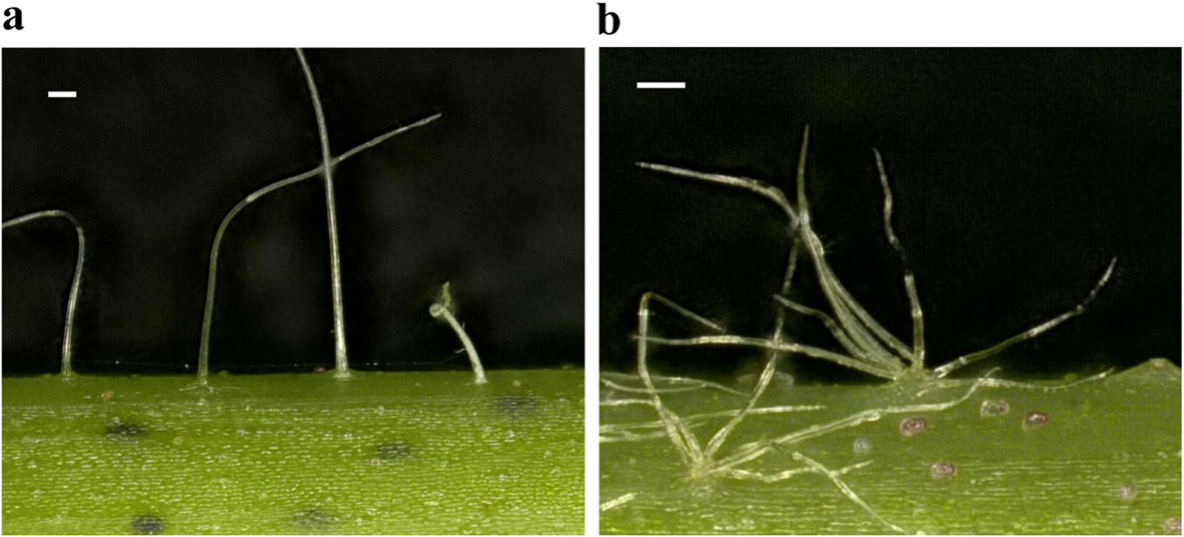
Two kinds of cotton stem trichomes from trichopores. **a** Single trichomes; **b.** Tufted/complex dendritic trichomes. Bars = 100 μm

**Fig. 2:**
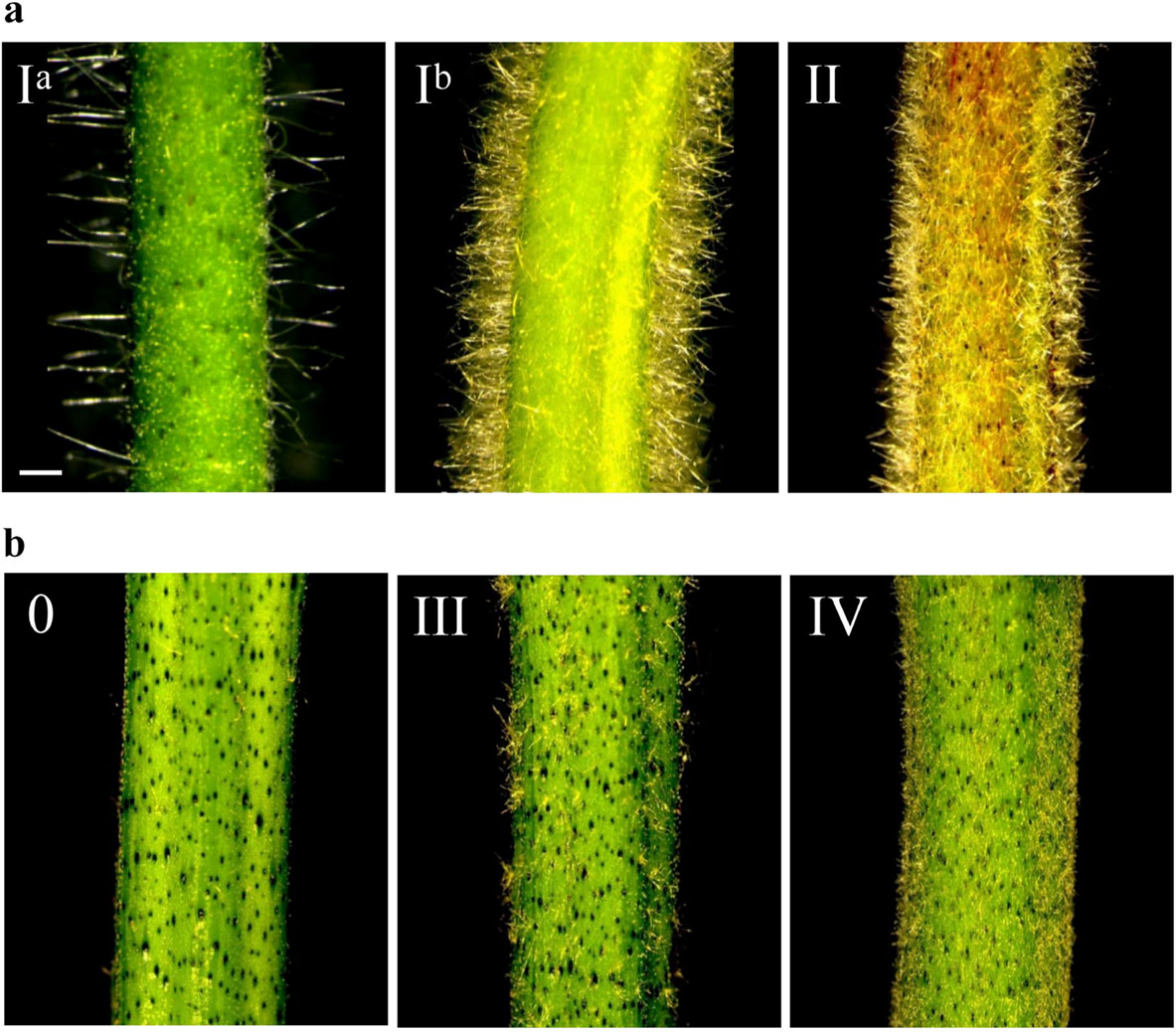
Stem trichomes observed using a stereo microscope. **a** Representative types of *G. hirsutum* trichomes: type I^a^ low-density (left, TM-1), I^b^ high-density (middle, NLD84), and type II trichomes (right, T586). **b** Representative types of *G. barbadense* trichomes: glabrous, type III, and type IV stem trichomes (from left to right, H7124, Aken 4154, and L-7009, respectively). Roman numerals in the top left corners are trichome types

There were two kinds of trichomes grown from trichopores, single and tufted/complex dendritic, as seen under a stereo microscope (Fig. 1), and they are similar to those from leaves [23]. The single trichomes were normally long and thin with sharp ends, while the tufted trichomes were normally shorter and grew from the same trichopore. The trichome numbers per trichopore varied from two to more than 10. Different cotton plants, even from the same species, differed in trichome number per trichopore, trichome density, and trichome length, forming clearly different trichome phenotypes. Stem trichome densities were grouped into six classes (0–5) from none to pilose (Fig. S2). As a whole, the cotton stem trichomes were categorized into four types (I–IV) (Table 1, Fig. 2). In Type I, most were singular long trichomes per trichopore, plus a few clusters, and the trichome density differed among different varieties. The stems with low trichome density levels (grades 1 and 2) were further classified as Type I^a^ and those with high trichome density (grade 4) as Type I^b^. In Type II, both singular and clustered trichomes occurred. Trichomes were shorter and coarser than those in Type I, with higher density resulting in a pilose phenotype over the whole plant. In Type III, multiple trichomes formed clusters from one trichopore. They grew sparsely on stems and were shorter than those of Type II. In Type IV, the tufted/complex dendritic trichomes were extremely short and dense, looking as if they were covered by a layer of powder.

**Table 1:** Classification of trichome phenotypes on stems of cultivated cottons

Trichome type	*Gh/Gb*^a^	Density	Clustered/single	Length
Type I	*Gh*	Low to high	Single	Very long
Type II	*Gh*	Very high	Single+clustered	Short
Type III	*Gb*	Low to median	Clustered	Very short
Type IV	*Gb*	Very high	Clustered	Extremely short

^a^ *Gh*, *G. hirsutum*, *Gb*, *G. barbadense*

**Fig. 3:**
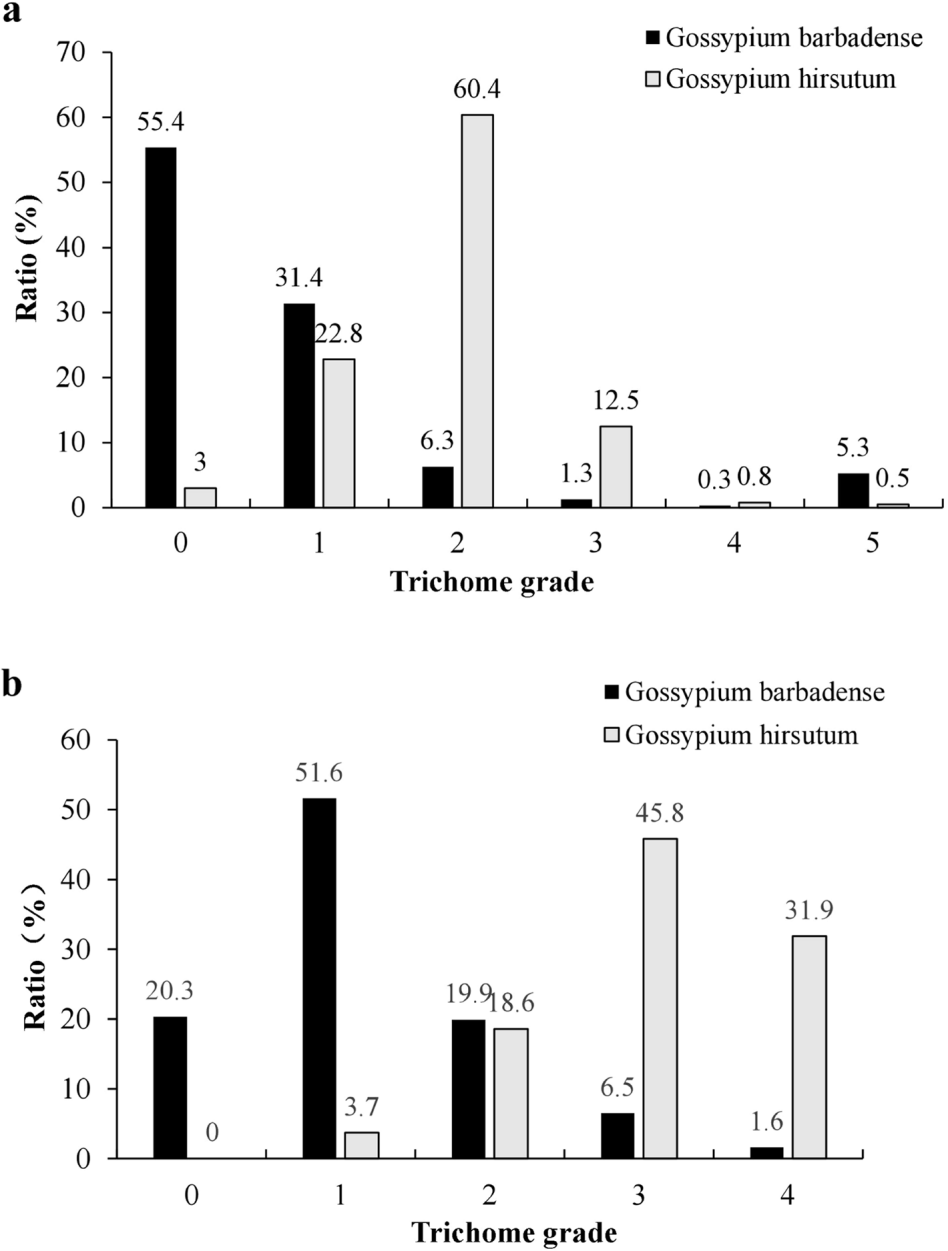
Frequencies of different trichome types (grades 0–4) in two cultivated cottons. **a** Data were downloaded from COTTONGEN (https://www.cottongen.org/), stem trichome information for individual varieties was kept in Table S6 and https://www.cottongen.org/find/qualitative_traits. **b** Data was from this experiment. Individual variety’s stem trichome information was kept in Table S1 and S4

The trichome phenotypic data of 11,442 *Gh* and 4323 *Gb* varieties/accessions were downloaded from the USDA National Cotton Germplasm Collection, one of the largest collections of cotton germplasm resources [24] (Table S6), and used to determine the percentages of cultivars having different stem trichome densities. The two species differed significantly in the proportions of glabrous varieties (Fig. 3a). In total, 55.4% of the *Gb* varieties (accessions) did not grow or had very few trichomes on their stems, while 97% of the *Gh* varieties had trichomes in grades 1–5, and only 3% had glabrous stems. In addition, the two cultivated tetraploid cottons were clearly different in all aspects of the trichome phenotype. Type I was the main type on *Gh* stems, accounting for 95% (Table 1, Fig. 2), while Type III was the main type on *Gb* stems, accounting for 31.4%. In addition, 5.6% of *Gb* varieties had Type IV trichomes (grades 4 and 5), while less than 1% of *Gh* had Type II trichomes (grades 4 and 5). Similar results were obtained from 295 *Gh* and 246 *Gb* varieties (Table 2, Fig. 3b, Table S1 and S4). Therefore, the two cultivated species had distinctive stem trichome phenotypes, especially their trichome density levels and trichome types.

**Table 2:** Statistical analysis of the rates of different trichome densities in sub-groups of *G. hirsutum*

*G. hirsutum* genotypes	Population size	Fuzzless rate (%)	Trichome density^a^				
			0	1	2	3	4
Fuzzless mutants	37	100	1.0	20.0	12.0	3.0	1.0
			(2.7)	(54.1)	(32.4)	(8.1)	(2.7)
Wild accessions (Late-maturing)	81	28.4	27.0	10.0	24.0	15.0	5.0
			(33.3)	(12.3)	(29.6)	(18.5)	(6.2)
Wild accessions (Normal-maturing)	99	26.3	4.0	15.0	29.0	38.0	13.0
			(4.0)	(15.2)	(29.3)	(38.4)	(13.1)
Modern varieties	295	1.4	0.0	11.0	55.0	135.0	94.0
			(0.0)	(3.7)	(18.6)	(45.8)	(31.9)

^a^Numbers in parentheses represent the percentages of plants within the different sub-groups of trichome density

### Wild *G. hirsutum* accessions differed significantly in stem trichome morphology from modern cultivated varieties

We observed the stem trichome phenotypes of wild *Gh* accessions planted in Hainan, China (Table S2) and found significant differences between wild forms and modern varieties. Compared with modern upland cotton, the wild accessions showed the following trichome characteristics (Table 2, Table S2 and Fig. S4): (1) No or fewer trichomes on stems. Among 180 wild accessions of seven wild races, 30 (16.8%) were glabrous, while only about 3% of the modern varieties in the data from the USDA National Cotton Germplasm Collection (Fig. 3) and none of the varieties we collected were glabrous (Table 2, Table S1). When stem pubescence was compared among the wild accessions based on flowering time, those that flowered later had a greater proportion of glabrous accessions than those that flowered at regular time as modern varieties. Among 81 late-flowering accessions, 37 (45.6%) were glabrous, or had grade 1 trichomes, whereas among 99 regular flowering accessions, only 19 (19.2%) were glabrous, or had grade 1 trichomes (Table 2). When different wild races were compared, race marie-galante had more glabrous accessions than the other wild races. (2) A large number of wild accessions had shorter and denser trichomes than modern varieties. Among the 149 accessions having stem trichomes, 66 (44.3%) were shorter than the Type I modern varieties, including 28 similar to Type II and 6 that were even shorter, having the pilose phenotype (Table S2, Fig. S4), especially among the *morrillii* race. As a whole, wild *G.hirsutum* accessions either have more ratio of glabrous stem, or more ratio of shorter trichome compared to modern varieties.

### Stem trichome phenotypes of diploid ancestors of tetraploid cotton

**Fig. 4:**
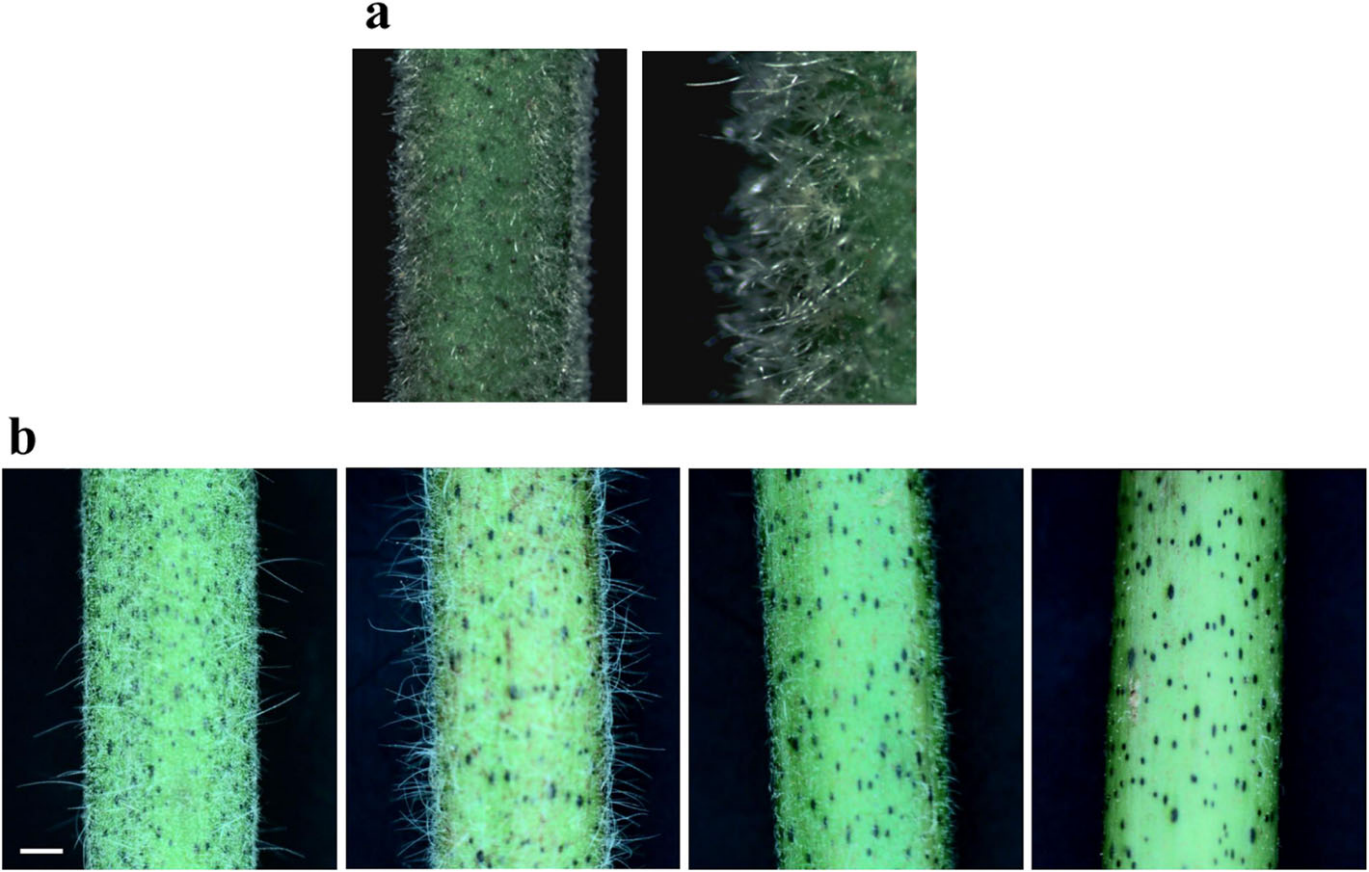
Stem trichomes of *G. raimondii* (**a**) and *G. arboreum* (**b**)

To compare the stem trichome phenotypes of diploid ancestors with those of cultivated tetraploid cottons, 77 individual plants from 12 accessions of *G. arboreum* (Table S7) and 5 individual plants from *G. raimondii* were observed. All the *G. raimondii* plants had very short and dense clustered trichomes on their stems, similar to *Gb* Type IV (Fig. 4a). Compared with *G. raimondii*, *G. arboreum* had more variation in stem trichome phenotypes (Fig. 4b). As in *Gh*, *G. arboreum* accessions also had singular and clustered trichomes. However, the clustered trichomes of *G. arboreum* were shorter than those of *Gh* and even shorter than *Gb* Type IV. As a result, the clusters were difficult to visualize. On the basis of their growth situations, the stem trichome phenotype of *G. arboreum* was classified into four types: single plus cluster, only single, only cluster, and glabrous. The stem trichome phenotypes of 12 *G. arboreum* accessions were investigated. Six had singular and clustered trichomes, four only had clustered trichomes, one only had singular trichomes, and one had the glabrous phenotype. Two diploid ancestors differ significantly. *G. raimondii* has the similar stem trichome as *Gb*, while *G. arboreum* has the stem trihomce like *Gh*.

### Genetic mapping of stem trichomes

To reveal the genetic basis regulating different types of stem trichomes, five crosses involving varieties having different types of stem trichomes were made (Table S5). Cotton stem trichome initiation is believed to be mainly controlled by genes on Chrs. 06 and 24 [15, 16, 22, 25, 26]. Therefore, our mapping efforts focused on these two genes and 77 polymorphic DNA markers, 23 on Chr. 06 and 49 on Chr. 24, among them 54 were newly developed based on reference genome sequences of *G. hirsutum* var. TM-1 [20, 27] (Table S8). Definitely, it is an efficient way to find the gene(s) related to trichome phenotype, but also will miss some other loci.

#### Type I trichomes in *Gh*

**Fig. 5:**
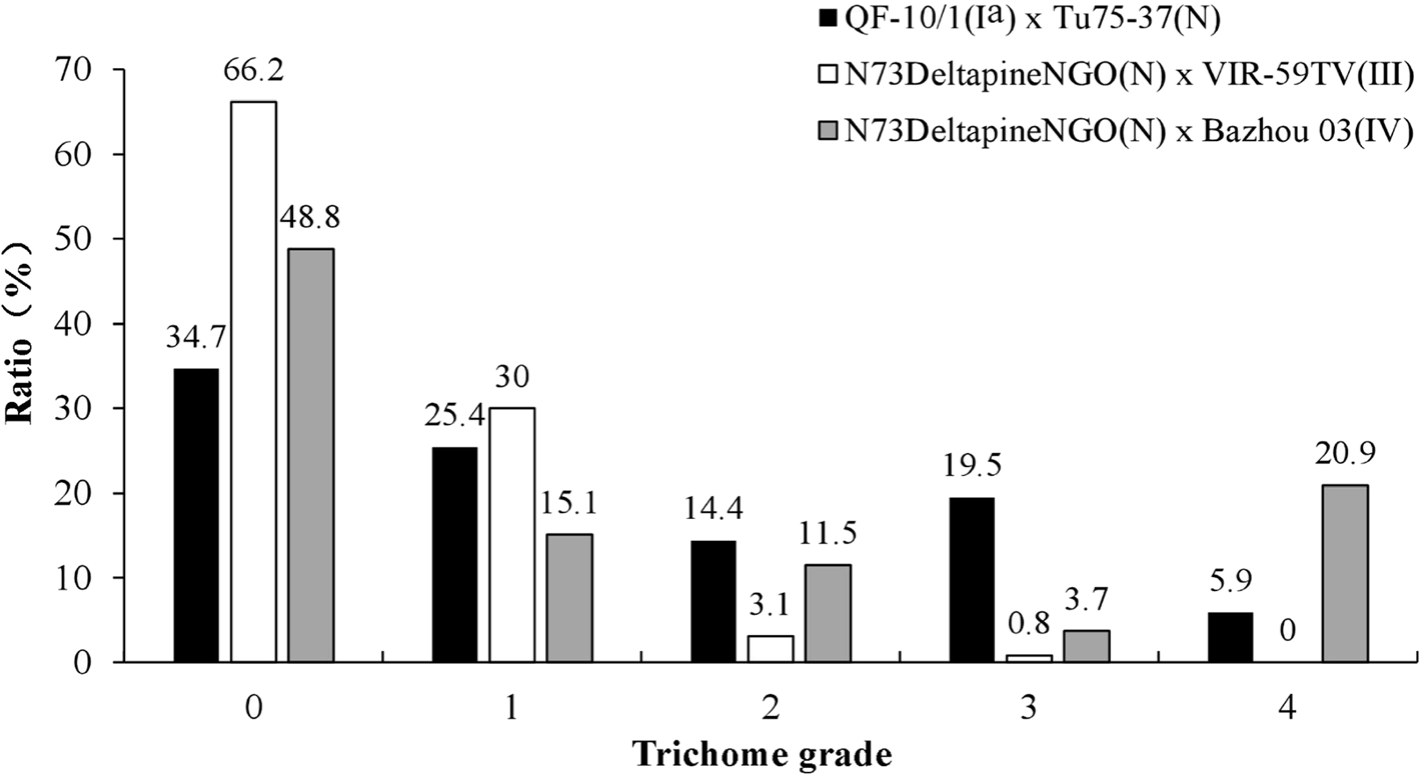
Ratio of plants having different stem trichome density levels in three F_2_ populations derived from crosses involving varieties having Type I^a^, III, and IV stem trichomes

**Fig. 6:**
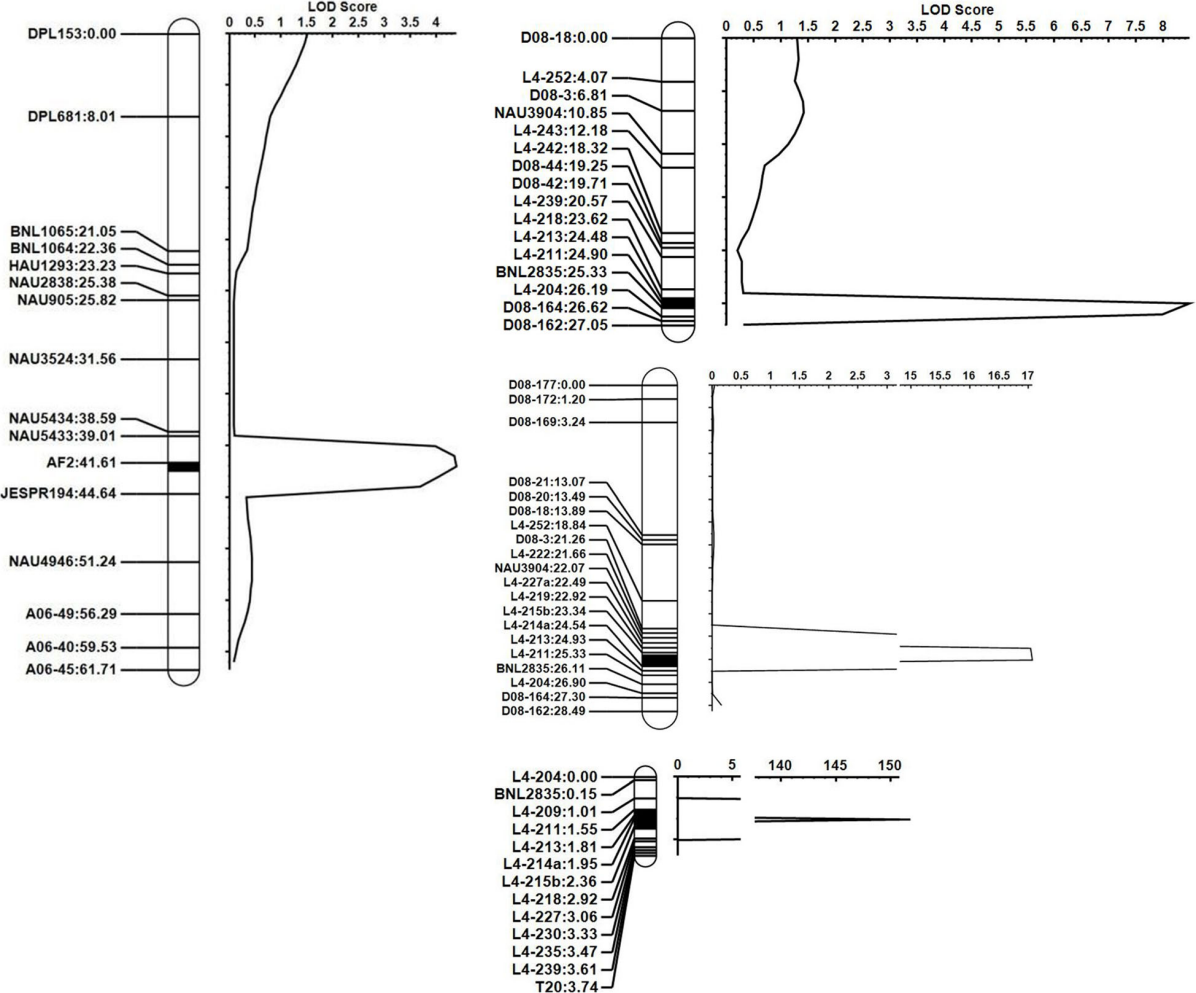
Genetic maps of QTLs related to type I^a^, III, and IV stem trichomes. **a** Type I^a^ on Chr.06; **b** Type I^a^ on Chr.24; **c** Type III on Chr.24; **d** Type IV on Chr.24. Some co-segregation makers were not shown in the figure, but listed in the Table S8

QF-10/1 is a *Gh* variety with Type I^a^ stem trichomes, grade 2, similar to genetic standard line TM-1. To map the genes conferring this type of stem trichome, QF-10/1 was crossed with Tu 75–37, a glabrous *Gb* variety. No trichomes grew on the stems of the F_1_ plants. An F_2_ population consisting of 118 plants showed continuous variation, and most of them (60%) had grade 0 or I trichome, while some had grade IV trichomes, surpassing the *Gh* parent (Fig. 5). Using this population, genetic maps were constructed for Chrs. 06 and 24, consisting of 18 and 24 DNA markers, respectively (Fig. 6a, b, Some co-segregation markers were not displayed in the figures. All marker information was listed in the Table S5. The trichome grade and genotypes of the F_2_ plants were given in Table S11). Combining the trichome phenotyping and genotyping data from this population, a QTL that had a LOD value of 4.4 and explained 18.4% of the phenotypic variation was detected in the region between NAU5433 and JESPR194 on Chr. 06, and was tightly associated with AF2, a SSCP marker developed by the *GhHD1* sequence (Table 3, Fig. 6a, Table S8). Another QTL was detected in the region between D08–163 and L4–239 on Chr. 24 that had a LOD value of 8.6 and explained 30.6% of the phenotypic variation (Table 3, Fig. 6b).

**Table 3:** Mapping information of QTLs associated with types I, III and IV stem trichomes

Cross combinations^a^	Chromosome	Left Marker	Right Marker	LOD	Add	PVE(%)
QF-10/1(I^a^) × Tu 75–37(N)	6	NAU5433	JESPR194	4.4	0.69	18.4
	24	D08–164	L4–239	8.6	1.09	30.6
N73DeltapineNGO(N) × VIR-59TV(III)	24	L4–211	NAU3904	17.6	−0.52	51.5
N73DeltapineNGO(N) × Bazhou 03 (IV)	24	L4–209	T10	149.4	−1.87	83.7

^a^letters in the brackets represented different stem trichome type. N means no trichome on stem. Position of some makers in the genetic map can be found in Table S8

#### Type II trichomes in *Gh*

Previously, we mapped the Type II stem trichomes of T586 to Chr. 06 and confirmed that its pilose phenotype was probably controlled by the *HD1* gene, using an F_2_ population derived from the cross between T586, a typical *Gh* genetic standard line with grade 5 stem trichomes and Pima S6 with glabrous stems [22]. To further clarify the genetic relationship between Type I and II stem trichomes, we crossed the *Gh* variety Liao1779, having a pilose morphology and grade 5 stem trichomes like T586, with Xinyan96–48, a variety having Type I^a^ stem trichomes (grades 1 and 2). The F_1_ plants had stems similar to those of Liao1779, covered with heavy trichomes. In total, 66 F_2_ plants showed clearly different trichome densities, most (52) having a high density of trichomes like Liao1779 (grades 4 and 5), and 11 had stem trichomes like Xinyan96–48, grades 1 and 2. Only three plants had intermediate trichome density levels (Table S9). These results indicated that the pilose stem (Type II) was probably controlled by a dominant gene. Using AF_2_, we found that both parents showed very good polymorphism, and the segregation of stem trichome density in this F_2_ population was closely correlated with the genotype of the SSCP marker (Table S9), suggesting that the pilose stem trichomes of Liao1779 were also controlled by *GhAt-HD1*, as in T586 [22].

#### Type III trichomes in *Gb*

This type had sparse clustered trichomes on the stems, grading 0 or 1. To map this type of trichome, N73DeltapineNGO, a *Gh* variety with glabrous stems, was crossed with VIR-59TV, a *Gb* variety having the typical Type III stem trichomes. The F_1_ did not have, or had only a few trichomes on stems, like the *Gh* parent. In the F_2_ population, most plants (86 of 130) did not have, or had only a few trichomes, like the F_1_ plants. Approximately 1/3 of F_2_ plants had stem trichomes of grade 1, and a few were graded at > 2 or 3 (Fig. 5). Thus, the glabrous stem of N73DeltapineNGO is probably controlled by a dominant gene. To map this gene, a genetic linkage map of Chr. 24, consisting of 28 markers, was constructed, and a QTL in the region between L4–211 and NAU3904 that had a LOD value of 17.6 and explained 51.5% of phenotypic variance was identified (Table 3, Fig. 6c. The trichome grade and genotypes of the F_2_ plants were given in Table S12).

#### Type IV trichomes in *Gb* varieties

Bazhou 03 and L-7009 had typical Type IV stem trichomes. To map the genes correlated with this phenotype, Bazhou 03 was crossed with N73DeltapineNGO. The F_1_ plants showed a trichome phenotype similar to that of N73DeltapineNGO. The F_2_ plants showed continuous segregation in trichome density, but displayed two peaks at grades 0 and 4 (Fig. 5). Among 383 F_2_plants, 244 (63.9%) had few trichomes, graded 0 and 1, similar to N73DeltapineNGO; and 80(20.9%) had many trichomes, graded 4, similar to Bazhou 03. Only 44 (11.5%) plants had trichomes of grade 2, and very few plants had trichomes of grade 3. When a trichome density of less or equal to grade 2 was used to form a group and a trichome density greater than grade 2 formed a second group, the ratio of the two groups of plants was close to 3:1, again suggesting that the glabrous stem trichomes of N73DeltapineNGO were probably controlled by a dominant gene. To map this gene, 21 polymorphic SSR and SSCP markers were used to genotype the F_2_ plants, and a linkage map covering 3.74 cM was constructed (Fig. 6d. The trichome grade and genotypes of the F_2_ plants were given in Table S13). A significant QTL having a LOD value of 149.1 and explaining 83.7% of the phenotype variation was detected in the region between L4–209 and T10 (Table 3, Table S8). All these mapping results strongly suggested that the gene on Chr. 24 is essential in regulating stem trichome initiation.

To reveal the genetic relationship between the Types III and IV stem trichomes of *Gb* varieties, Aken 4154 (Type III) was crossed with Bazhou 03 (Type IV). The F_1_ plants showed a trichome phenotype similar to that of Aken 4154, with a few clustered trichomes, and some had almost no trichomes. Like the (Bazhou 03 x N73DeltapineNGO)F_2_ population, this F_2_ population showed clear segregation in stem trichome density (Table S10). Among 64 F_2_ plants, 44 had stem trichomes of grades 0 and 1, whereas 18 had stem trichomes of grades 3 and 4, and only two had trichomes of grade 2. This result again suggests that the pilose phenotype of Bazhou 03 was controlled by a recessive gene. A polymorphic SSR marker (D08–98, Table S8) which had a very close correlation with stem trichome phenotype (Fig. 6c) was used to genotype the F_2_ plants, also revealing a close correlation with stem trichome density, with only three exceptions (Table S10), suggesting that Types III and IV stem trichomes in *Gb* were controlled by the same gene but different alleles, and that Type III was dominant to Type IV. Above mapping efforts revealed that the different types of *Gh* and *Gb* stem trichome were mainly controlled by different combinations of genes or alleles on Chr.06 and 24. The one on Chr.24 probably plays even more important function compared to one on Chr.06, especially in *Gb*.

### Inter-specific and intra-specific variations in stem trichome phenotypes **are** reflected in F_1_ hybrids of crosses between *Gh* and *Gb* varieties having different types of stem trichomes

To further clarify the classification of stem trichomes, more inter-specific crosses between *Gh* and *Gb* varieties with different types of stem trichomes were performed (Table 4). Interestingly, when *Gh* and *Gb* varieties having the same type of stem trichomes were crossed, the F_1_ hybrids always showed a similar stem trichome morphology (Table 4, Fig.S5). However, F_1_ plants from crosses between *Gh* and *Gb* varieties with different types of stem trichomes always displayed different stem trichomes (Table 4, Fig.S5). For example, when *Gh* varieties with Type I^a^ stem trichomes were crossed with *Gb* variety H7124, a representative of the glabrous varieties, the F_1_ hybrids did not grow trichomes on their stems. However, when these *Gh* varieties were crossed with Aken 4154 or L-7009, representatives of *Gb* varieties with Types III and IV stem trichomes, respectively, the resultant F_1_ always had stem trichome phenotypes close to Types III and II, respectively (Table 4, Fig.S5). These results suggested that the stem trichome alleles in *Gb* are dominant to those of *Gh* Type I^a^ trichomes, except for those responsible for the lengths of the F_1_ hybrids of Type I × Type IV, which were intermediate between the two parents. When three *Gh* varieties with Type I^b^ stem trichomes were crossed independently with the above three *Gb* varieties, most of the F_1_ plants showed different stem phenotypes from the above crosses with the same *Gb* varieties. When *Gh* varieties with Type II trichomes were crossed with H7124 and L-7009, the F_1_ plants had a stem trichome phenotype similar to Type II of *Gh*, indicating that *Gh* trichome genes are dominant to those of *Gb*. These results further confirmed that classification of stem trichome established in this study was correct and can be further used in gene cloning and cotton breeding.

**Table 4:** Stem trichome phenotypes of F_1_ plants from crosses between *G. hirsutum* and *G. babradense* varieties having different types of stem trichomes

Trichome type, genotype (Gh, Gb)		0	III	IV
		H7124	Aken 4154	L-7009
I^a^	TM-1	0	III, 1	II, 4
	T Kuo	0	III, 2	II, 4
	JS1	0	III, 1	–
	NLD76	0	III, 2	II, 4
	9754–1	0.5	III, 1	II, 4
I^b^	9741–3	III, 1–2	–	IV, 4
	XLZ53	III, 2	III, 4	II, 4
	NLD68	III, 1	III, 3–4	IV, 4
II	NLD56	II, 4	–	II, 4
	T586-M	II, 4	–	II, 4

Roman numbers represent the trichome types. Arabic numbers represent trichome density. − indicates that the data are not available

### Stem trichome density is closely related to fiber initiation and development in both *G. hirsutum* and *G. barbadense*

We analyzed the correlation between the densities of fuzz fibers and stem trichomes. First, we examined the fuzz fibers of *Gh* wild species, which showed significant differences in stem trichome phenotypes compared with modern varieties. A much greater proportion of them had no or fewer fuzz fibers than the modern varieties (~ 27% vs 1.4%, Table 2, Table S2), which suggested a correlation with trichome density. In addition, more wild species had shorter and denser stem trichomes than modern varieties, which was also in accordance with fiber length, because most wild *Gh* accessions have very short lint fibers. Thus, fuzzy/lint fibers and stem trichomes may have at least partly overlapping genetic bases for regulating their initiation and development.

To further investigate the relationship between stem trichomes and fiber densities, we observed the stem trichomes of 37 *Gh* fuzzless mutants and found that there was a much higher percentage of the mutants having no or few trichomes (grades 0 and 1) than in the common varieties (56.8% vs 3.7%) (Table 2, Table S3). Only 11% of the fuzzless mutants had denser trichomes (grades 3 and 4), compared with the common varieties (78%).

As indicated above, most *Gb* varieties did not have, or had very few, stem trichomes. The presence of naked seeds in most *Gb* varieties was another characteristic that differed from the *Gh* varieties. Out of 121 *Gb* varieties, 61 (50%) had less fuzz fiber on seeds (fuzz fiber density less than 2) than the modern *Gh* varieties. An association analysis revealed very close correlation between the densities of stem trichomes and fuzz fibers (*r* = 0.501**, *P* < 0.05) (Table S4), suggesting that the genetic foundation of *Gb* stem trichome development was at least partially the same as that of fibers found in *Gh*.

## Discussion

### Stem trichome development had simpler genetic control than leaf trichome

Trichomes, as part of the outermost layer of the plant body, are important for resistance to biotic and abiotic stresses. The molecular mechanisms of their growth, as a unique model, have been investigated extensively, including their morphology and botanical genesis, especially in *Arabidopsis* [28]. Cotton trichomes have received even more attention than most, owing to similarity to seed trichomes (fibers, the primary economic product) in growth and evidence that these organs have an overlapping genetic basis. A classical genetic study indicated that cotton pubescence (mostly on leaf trichomes) was mainly controlled by five loci (*t*_*1*_*–t*_*5*_) [29]. A corresponding allelic series was renamed *T*_*1*_, *T*_*1*_^*h*^, *T*_*1*_^*to*^,and *T*_*1*_^*an*^ [29].*T*_*1*_ on Chr. 06 is the main locus for whole-plant pubescence, including stem trichomes [13, 14].

Genetic mapping using DNA markers allocated the loci for leaf pubescence onto five chromosomes. The major locus, recognized as *t*_*1*_, was mapped to the centromeric region of Chr. 06, and the other four (*t*_*2*_*–t*_*5*_) were mapped to chromosomes 01, 07, 23, and 25, respectively [15]. Interestingly, only one locus for stem trichomes was detected, on Chr. 23. In another study [25], only the *T*_*1*_locus for leaf pubescence was again found in the same region of Chr. 06. Seven QTLs for leaf trichomes were mapped to different chromosomes: LGA01 (Chr. 13), Chr. 17, and LG. D03 (Chr. 24) [25]. Only two QTLs for stem pubescence were mapped, to Chr. 06, the same as for leaf trichomes, and Chr. 24, respectively. Thus, three main loci for stem pubescence were identified, fewer than for leaf pubescence, and only *T*_*1*_ on Chr. 6 is also essential for leaf pubescence [16, 22, 25].

**Fig. 7:**
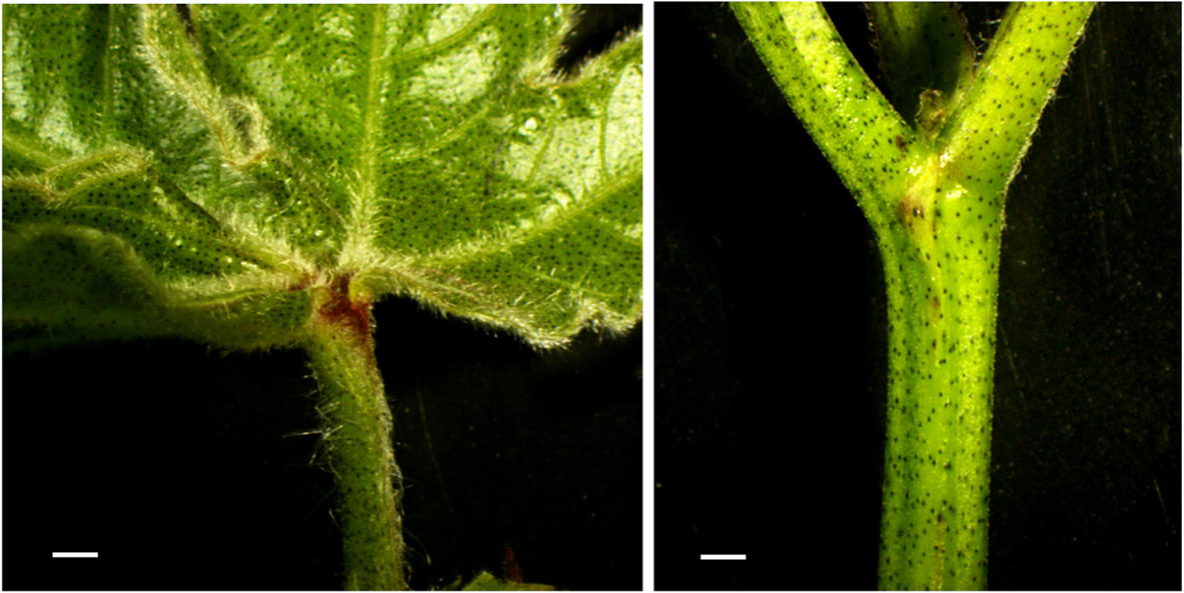
Pubescent leaf and glabrous stem of *G. hirsutum* wild race Palmeri TX-879b

Many *Gh* varieties/wild species have glabrous stems, but pubescent leaves (Fig. 7). The glabrous stems of *Gh* varieties and sparse trichomes (Type III) of *Gb* varieties were confirmed here to be controlled by a dominant gene on Chr. 24, consistent with an earlier report [30]. These results suggest that stem trichome initiation has a simpler genetic foundation than leaf pubescence, with one major locus shared with leaves, and additional factors. Different leaf parts (petiole, margin, vein, and others) can have different numbers of trichomes, that can be mapped as different trichome traits [15], making the research more complicated, but there were no such variations for stems. In addition, stem trichome density was closely correlated with fuzz fiber density. The results suggested that stem trichomes are a very good model for investigating genetic mechanisms that regulate fiber development.

### The stem trichome development of two cultivated tetraploid cottons was governed by different gene/allele combinations and experienced different evolutionary fates during domestication

A clear difference between *Gh* and *Gb* was reported in the molecular mapping of trichome traits, especially for stem trichomes, although their mapping populations were derived from crosses between *Gh* and *Gb* varieties [15, 25]. In the present study, *Gh* and *Gb* stem trichome phenotypes were classified into four types. *Gh* varieties normally had Types I or II trichomes, while *Gb* had Types III or IV trichomes. The F_1_ plants generated by crossing varieties having the same type of stem trichomes always showed a similar trichome phenotype, and those from varieties having different types of stem trichomes showed various stem trichome phenotypes, suggesting that the different types of stem trichomes are controlled by different genes or allele combinations. *Gb* varieties with glabrous stems and Type III trichomes were similar in phenotype, because they have no and few clustered trichomes, respectively. The glabrous stems were confirmed to result from a transposable element (TE) insertion in *HD1*, a candidate gene of *T*_*1*_, while this gene in cotton having Type III stem trichomes does not have such an insertion (Ding et al. 2015). This phenotype was confirmed to be caused by the gene on Chr. 24 in this study. The *Gb* parent Pima-S7 used by Wright et al. (1999) was confirmed to carry a TE insertion in *HD1*, while *Gb* variety VH8–4602 used by Lacape and Nguyen (2005) was inferred as having Type III trichomes, because several trichomes (0.33) appeared on the stems. These findings not only explain why different studies produced different mapping results for genes related to stem and leaf trichomes, but also support a previous conclusion [26] that similar genetic and molecular mechanisms regulate stem trichome initiation in both *Gh* and *Gb*. However, they have undergone significant levels of differentiation during their speciation.

Through genetic mapping using different cross combinations, Type I trichomes were found to be controlled by genes on Chr. 06 and Chr. 24, Type II by a gene on Chr. 06, and Types III and IV by a gene on Chr. 24. The allele on Chr. 06 of Type II is dominant to the corresponding Type I allele. In *Gb*, the allele on Chr. 24 for Type III was dominant to the allele for Type IV. Classic genetic mapping [15, 25] and a transgenic study [21], together with our current findings, indicate that *T*_*1*_ is a major locus involved in determining cotton stem trichome growth. Lacape and Nguyen (2005) first reported that the gene on Chr. 24 was probably another major locus for stem trichomes. The current study revealed that most glabrous stems of *Gh* wild accessions/cultivars and Type III stem trichomes of *Gb* varieties were controlled by a dominant gene on Chr. 24. Therefore, the genes on Chr. 06 and Chr. 24 are essential for determining the stem trichome phenotypes of the two tetraploid species. However, in the effort of mapping *Gh* Type I stem trichome, the genes on Chr.06 and Chr.24 only explain about 50% of phenotype variation, suggesting additional other genes, such as the one on Chr. 23 [15], exist regulating stem trichome development, and more efforts are required to fully understand their genetic mechanisms.

The whole-genome sequencing of the two cultivated tetraploid species revealed their genome differentiation during domestication and variety improvement at the DNA level, reflected by significant variations in plant morphology and fiber quality [20]. In our previous studies, we indicated that the two species differ greatly in the proportions of varieties with and without stem trichomes among primitive accessions and modern cultivars [26]. The glabrous stems of *Gb* are the result of TE insertions in the gene *HD1*, while *Gh* has other important genes in addition to *HD1*, probably including the one on Chr. 24, which cause glabrous stems. In our ongoing project, we found that the glabrous stems of most *Gh* wild accessions were caused by a SNP mutation in the promoter of the candidate gene (unpublished data). In addition, *Gh* wild accessions have more diverse kinds of stem trichome phenotypes than modern varieties. For example, more wild *Gh* accessions are glabrous, or have shorter and denser stem trichomes, compared with modern cultivars. Interestingly, these stem trichome phenotypes are closely correlated with naked seeds and short fiber length. Similarly, more diverse stem trichome types exist in the *Gb* wild accessions than in the modern varieties [22]. This information, coupled with our knowledge of the key genes regulating stem trichome growth on Chrs. 06 and 24, suggests a hypothesis for the evolution of *Gh* and *Gb* stem trichomes along with fibers. According to this hypothesis, the glabrous stems of *Gb* wild accessions were caused by the TE insertion in *HD1* on Chr. 06, while the glabrous stems of *Gh* wild accessions were mainly caused by gaining the function of the gene on Chr. 24 (unpublished data), except for a few also caused by TE insertions in *HD1* [26]. The close correlation between stem trichomes and fuzz fibers suggests that the former in modern varieties evolved after selection for fiber length. Thus, both trichomes and fibers evolved in parallel, and the current stem trichome phenotypes in modern tetraploid cottons are partly a result of selection for seed hairs during domestication and variety improvement.

### Stem trichomes and seed fibers are epidermal hairs that are regulated by similar genetic factors

The trichomes of *A. thaliana* and cotton fibers are single-celled structures of epidermal organs. Thus, *Arabidopsis* could serve as a model for elucidating the genetic mechanisms controlling cotton fiber development [31, 32]. Many genes were identified by homoeologous cloning and confirmed to be related to fiber development [2]. There has been long-standing interest in whether the genetics of simply inherited traits, such as trichomes in cotton, could increase our understanding of more complex processes, such as lint fiber development [31, 33]. Simpson (1947) [34] reported that the pilose (*T*_*1*_) trait, which results in short dense trichomes on the vegetative parts of upland cotton, was associated with short coarse fibers. Since then, pilose has been shown to be related to yield components and fiber quality [13, 14, 16, 29]. Later, the pilose (*T*_*1*_) trait was confirmed to be controlled by *HD1* on Chr. 06 [22].

While the present study and several others focus on genotypic correlations between stem trichomes and seed fibers associated with variation on chromosomes 6 and 24, appreciable evidence suggests that this correlation applies to additional genes across the genome. In the current study, the growth of *Gh* stem trichomes was very closely correlated with fuzz fibers in both wild accessions and fiber mutants. The *Gb* stem trichome density was closely correlated with fuzz fiber density. These findings supplement and complement an independent study in which106 lines with leaf and stem trichome variations were shown to often have altered lint fiber characteristics, supporting the hypothesis that there is considerable overlap in the sets of genetic factors acting in the development of these analogous organs [33]. Revealing general mechanisms and specific genes regulating the density of stem trichomes will lay a foundation for understanding the mechanisms regulating the density of cotton fibers. In addition, our work can provide the cotton breeders with an idea that they should consider stem trichome as useful phenotype index during their selection of progenies in conventional breeding.

However, there are some limitations in this research which need more attentions in the future studies. (1) Not enough *G. raimondii* plants were used in morphology observation, which will probably affect the correct judgment of real stem trichome phenotype *G. raimondii* is a perennial species growing in the tropical areas of low latitudes and only five plants were keptin the green house. After observation, we found that they are all healthy and show consistent stem trichome phenotype, suggesting that they can be used as the representatives of *G. raimondii* plants. More *G. raimondii* plants should be included in the future for further trichome investigation and comparison between two diploid ancestors.(2) Limited plant numbers were used in F_2_ populations of (Liao1779x Xinyan96–48) and (Aken4154 xBazhou03), being 66 and 64 plants respectively (Table S5), which can only provide rough stem trichome genotype/phenotype correlation of Type I vs Type II and Type III vs Type IV cottons. In the future work, large mapping populations could be developed to uncover the close genetic relationship between different types of stem trichome. (3) Not enough DNA markers were used to cover entire genome in mapping QTL/genes related to stem trichome development. As a result, it may miss the discovery of other important genes as discussed in the previous section. Additional work should be considered to discover all possible stem trichome QTL/genes. Once more newly important trichome development related genes were mapped and cloned, its molecular regulating network will be further clearly revealed.

## Conclusions

In this study, stem trichome phenotype of tetraploid cottons was classified into four types (I-IV) after systematical observation. Upland cotton normally have single (Type I) or single plus cluster (Type II) stem trichome, while Sea Island cotton have very short cluster trichome, and some have sparse (III) and other have very dense trichome (IV). Based on our findings together with other reports, stem trichome initiation is convinced to be controlled by simpler genetic foundation compared to leaf, mainly by the gene on Chr.06 and the one on Chr.24. Different types of stem trichome were mainly determined by combination of these genes/alleles. We also observed very close correlation between fuzzy fiber number and stem trichome density in both *Gh* and *Gb* primitive accessions and modern varieties. Based on above findings, we suggested that stem trichome evolved with seed fiber in parallel during domestication of cultivated tetraploid cottons.

## Methods

### Plant materials

*Gossypium* genotypes used for studying stem trichome density included 295 cultivated varieties, 180 wild accessions, and 37 fiber mutants of *Gh*; and 246 cultivated varieties of *Gb* (Table S1–4). All cotton materials were obtained from National Medium-term Gene Bank of Cotton in China. *Gh* wild accessions, *Gh* fiber mutants, and *Gb* cultivated varieties were also used for examining fuzz fiber density. The varieties used to produce the F_1_ hybrids, which were used to confirm the classifications of stem trichome types, and the F_2_ populations, which were used for mapping stem-trichome-related QTLs, are listed in Table S5. *Gb* varieties were planted in Shihezi, Xinjiang for trichome and fuzz fiber phenotype observation. *Gh* fiber mutants, *Gh* and *Gb* parents, and the F_1_ and F_2_ populations were planted in a farm at Zhejiang A&F University, Zhejiang Province, China. *Gh* varieties and wild races were planted in the National Wild Cotton Nursery, Sanya, China for fiber phenotype observation.

### Observation of trichome and fuzz fiber phenotypes

The observation and density grading of stem trichomes were carried out as described [26]. During the blooming period, the trichome phenotypes of the top and second internodes were visually observed twice by two different researchers in the field to determine the possible grades (Fig.S1). The internodes were cut and photographed using a KoPa® Microscope (M101) for further observation in the laboratory, and pictures of internodes with typical phenotypes were taken using a stereo microscope (OLYMPUS SZX10). Trichome phenotypes were classified into six grades (0–5) from the absence of trichomes to pilose based on trichome density (Fig.S2). The bolls on the middle fruit branches of the plants were harvested, and seeds were grouped into five types (0–4) based on the fuzz fiber numbers (Fig. S3). The association between phenotype and genotype was analyzed using correlation coefficients calculated by CORREL in Microsoft EXCEL at *P* < 0.05 according to the *t*-tests.

### Marker development

Total genomic DNA was extracted from young leaves of different genotypes, including varieties, parents and each F_2_ individual, using a modified CTAB method [35]. According to a *G. raimondii* genome sequence [36] and tetraploid cotton TM-1 genome sequence [27], two types of molecular markers, simple sequence repeats (SSRs) and single-strand conformation polymorphisms (SSCPs) (Table S6), were developed to map trichome-related genes and QTLs. Primers were developed using Primer Premier (http://www.premierbiosoft.com/primerdesign/) and synthesized by BGI (Shanghai, China). PCR of SSR and SSCP markers were performed in 15-μL reaction mixtures each containing 1 μL of genomic DNA (100 ng/μL) as template, 0.3 μL of each primer (10 mmoL/L), 0.15 μL of r*Taq* DNA polymerase (5 U/μL) (TaKaRa, Japan), 0.25 μL of dNTPs (10 mmoL/L), and 1.5 μL of buffer solution (10×). The reaction program consisted of one cycle at 94 °C for 5 min, followed by 30 cycles at 94 °C for 30 s, 55 °C (according to the Tm values of different primers) for 40 s, and 72 °C for 30 s, followed by a final extension at 72 °C for 10 min. The PCR products were separated on a 1.0% polyacrylamide gel in 1× TBE buffer. After electrophoresis, the gel was silver stained. Analysis of SSCPs was carried out according to a procedure reported previously [18].

### Map construction and QTL localization

All markers polymorphic between parents were initially used to survey the F_2_ populations for mapping of genes and QTLs associated with stem trichomes. Linkage analysis was carried out using JoinMap 4.0 and a LOD score of 3.0. The Kosambi map function [37] was used to convert recombination frequencies into map distances. QTLs were analyzed using the composite interval mapping function in the QTL Ici Mapping V3.3 software [38].

## Supplementary Information

**Additional file 1: **Supplementary Figs. S1 to S5 (**Fig. S1.** Observations of cotton stem trichome phenotypes; **Fig. S2.** Stem trichome grading; **Fig. S3.** Seeds having different fuzzy fiber density levels grouped into five grades from 0 to 4 (from left to right); **Fig. S4.** Stem trichome phenotypes of the *G. hirsutum* wild accessions showing the different lengths and density levels; **Fig. S5.** Stem trichome phenotypes of parents and their F_1_ hybrids from crosses between *G. hirsutum* and *G. babradense* varieties with different types of trichomes.

**Additional file 2: Table S1.** Cultivated varieties of *G. hirsutum* used in this research. **Table S2.** *G.hirsutum* wild accessions used for observation of stem trichome density and fuzzy fiber. **Table S3.** Fussy fiber mutants of *G. hirsutum* used for observation of stem trichome density and fuzzy fiber. **Table S4.** Stem trichome and seed fiber of *G. barbadense* varieties. **Table S5.** Crosses used in mapping and classification of stem trichome type. **Table S6.** Stem trichome phenotype from NCGC. **Table S7.** *G. arboreum* stem trichome phenotypes. **Table S8.** Sequences, mapping location and physical position of SSR and SSCP primer used in mapping stem trichome. **Table S9.** Population derived from cross between Xinyan96–48 and Liao1779 for mapping Type II stem trichome. **Table S10.** Stem trichome phenotype of (Aken4154 x Bazhou03)F_2_ plants and their genotypes detected by D08–98. **Table S11.** Stem trichome phenotype of (QF-10/1 x Tu75–37) F_2_ plants and their genotypes detected by DNA markers, which were used for mapping Type I trichome of *G. hirsutum*. **Table S12.** Stem trichome phenotype of (N73DeltapineNGO x VIR-59TV)F_2_ plants and their genotypes detected by DNA markers, which were used for mapping Type III trichome of *G. barbadense*. **Table S13.** Stem trichome phenotype of (N73DeltapineNGO x Bazhou03)F_2_ plants and their genotypes detected by DNA markers, which were used for mapping Type IV trichome of *G. barbadense*.

## Data Availability

All data generated or analyzed during this study are included in this published article [seethe supplementary information files].

## Abbreviations

*Gh*: *Gossypium hirsutum*
*Gb*: *G. barbadense*
SSR: Simple sequence repeats
SSCP: Single-strand conformation polymorphism
TE: Transposable element
*HD1*: Homeodomain-leucine zipper gene
