# Supplementary material for: Differentiation in the genetic basis of stem trichome development between cultivated tetraploid cotton species
Source: BMC Plant Biol. 2021 Feb 25;21:115. doi: 10.1186/s12870-021-02871-4 (PMC7905624; doi:10.1186/s12870-021-02871-4)
Supplement: Supplementary file 1 — Additional file 1: Supplementary Figs. S1 to S5 (Fig. S1. Observations of cotton stem trichome phenotypes; Fig. S2. Stem trichome grading; Fig. S3. Seeds having different fuzzy fiber density levels grouped into five grades from 0 to 4 (from left to right); Fig. S4. Stem trichome phenotypes of the G. hirsutum wild accessions showing the different lengths and density levels; Fig. S5. Stem trichome phenotypes of parents and their F1 hybrids from crosses between G. hirsutum and G. babradense varieties with different types of trichomes. [file 12870_2021_2871_MOESM1_ESM.pdf]

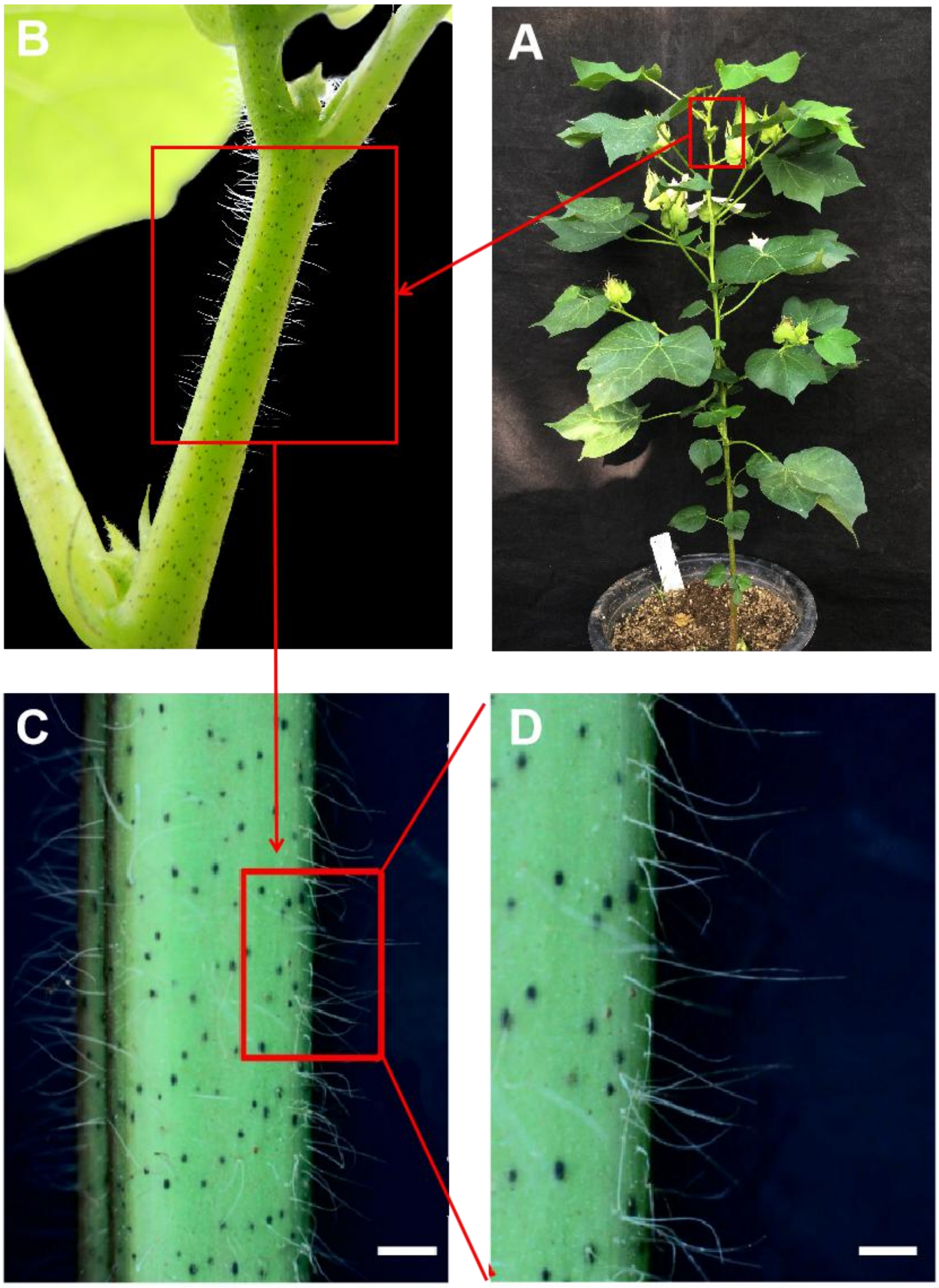

Fig. S1 Observations of cotton stem trichome phenotypes. **a.** Cotton plants at flowering; **b.** Top second internode; **c.** Micro-image of the top second internode; **d**.Magnified view of the cotton stem’s surface. Scale bars = 2 mm in **c** and 0.5 mm in **d.**

a

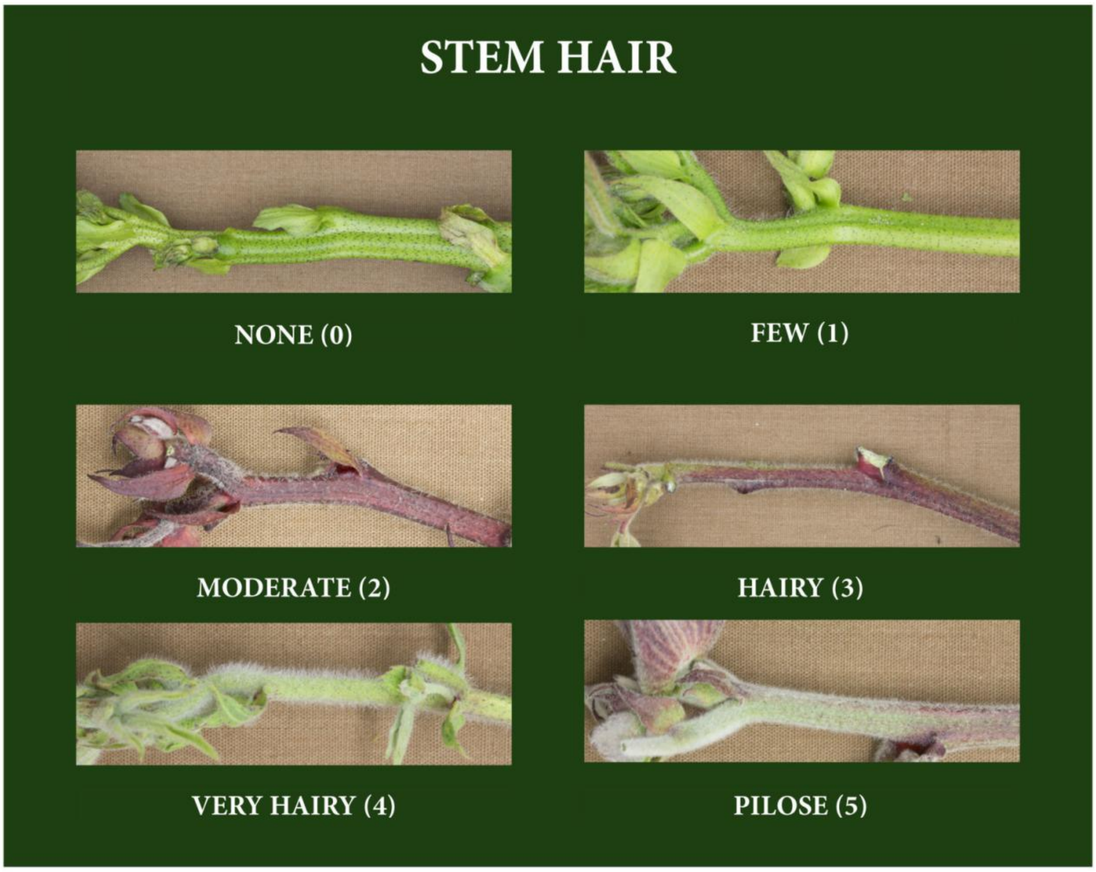

b

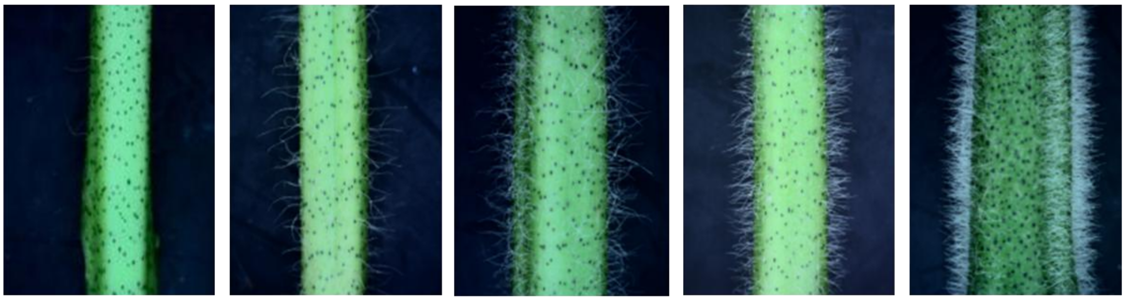

Fig. S2 Stem trichome grading. **a.** Stems with different trichome grades adopted from COTTONGEN (https://www.cottongen.org/); **b.** The five grades of stem trichome density used in this study (0-4 from left to right) . The numbers in both studies are almost consistent, with 0 representing glabrous (none) and 4 representing a very high density (very hairy).

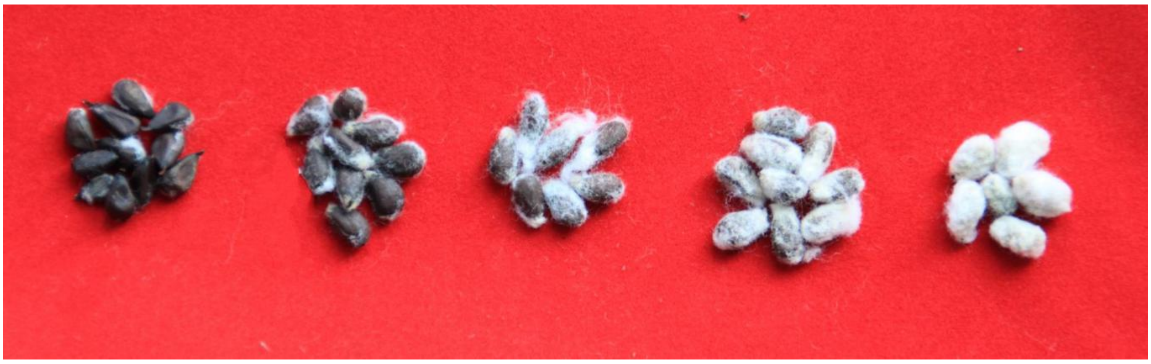

Fig. S3 Seeds having different fuzzy fiber density levels grouped into five grades from 0 to 4 (from left to right).

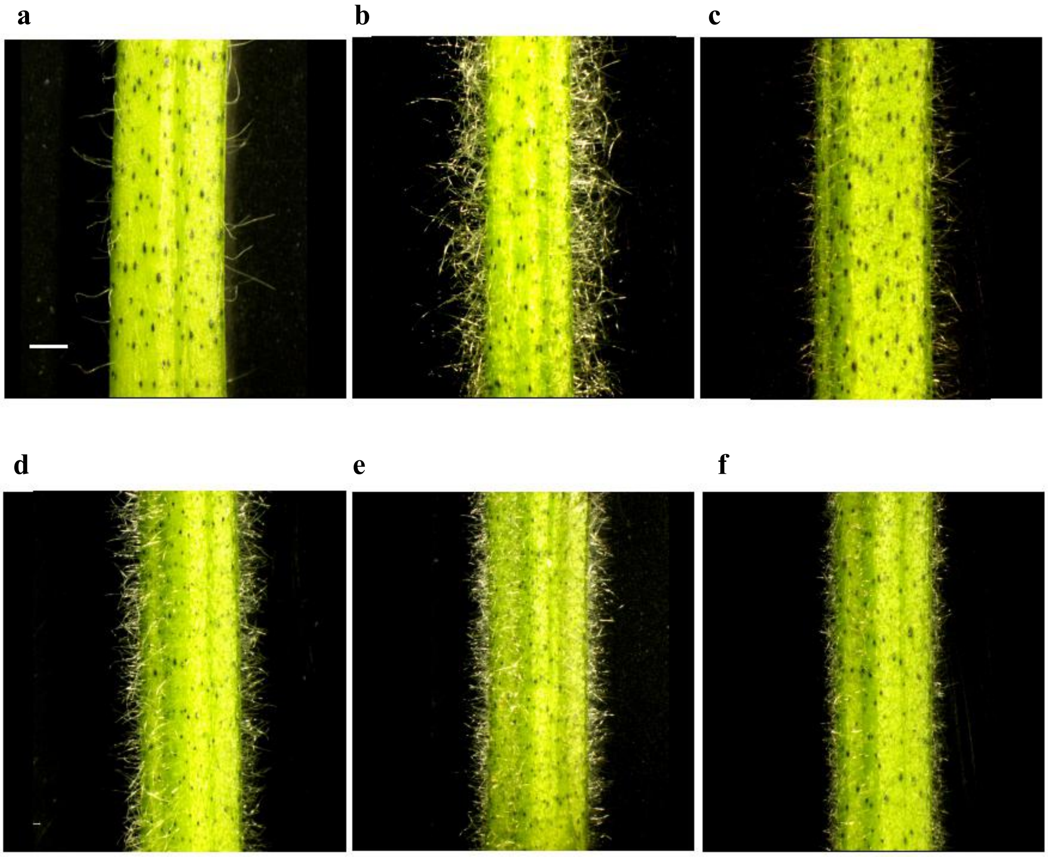

Fig. S4 Stem trichome phenotypes of the *G. hirsutum* wild accessions showing the different lengths and density levels. **a** Type I^{a}; **b** Type I^{b}; **c** Type I-II; **d** Type II; **e** Type II-IV; **f** Type IV. Bar = 1 mm.

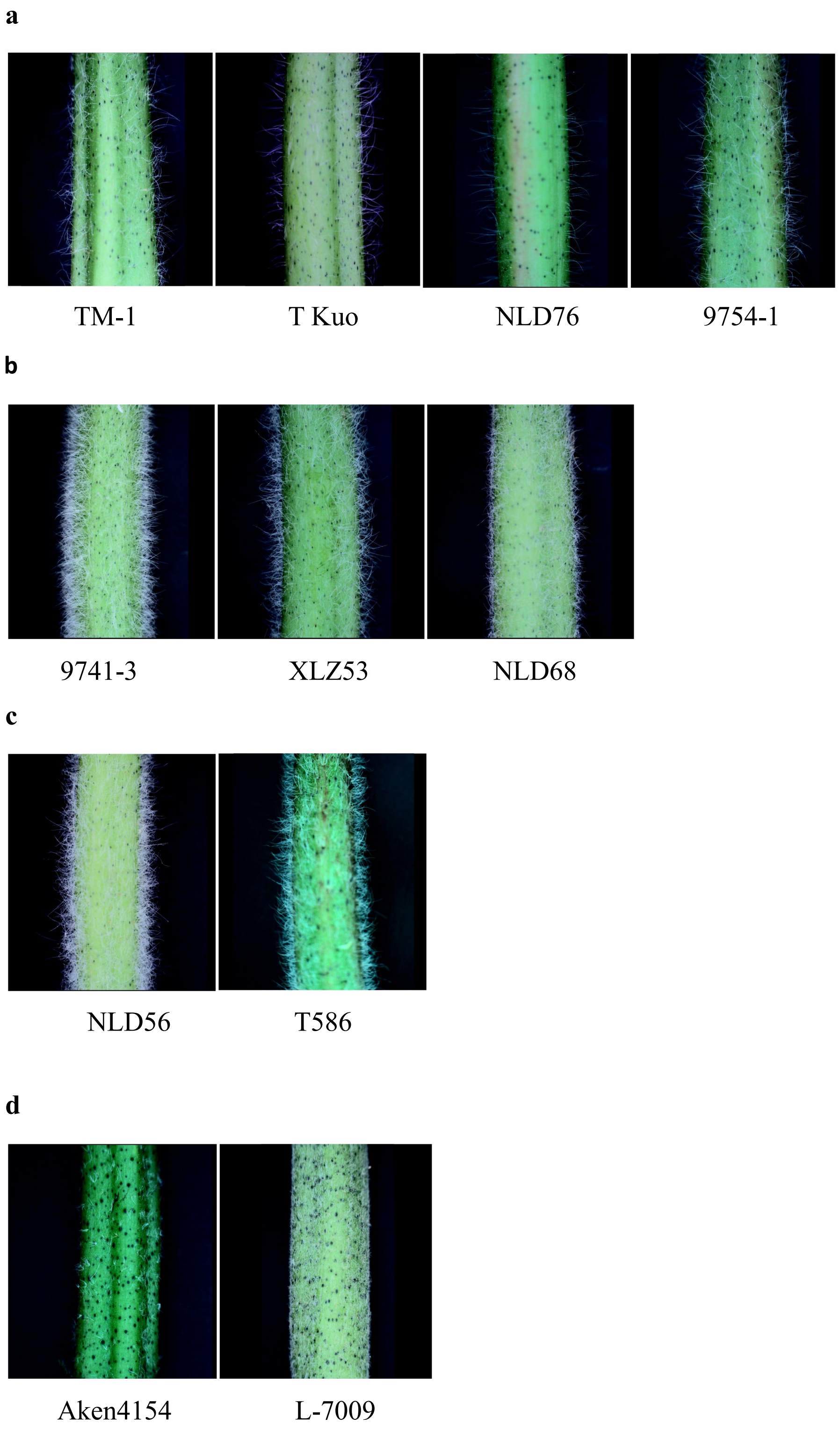
a
TM-1
T Kuo
NLD76
9754-1
b
9741-3
XLZ53
NLD68
c
NLD56
T586
d
Aken4154
L-7009

e

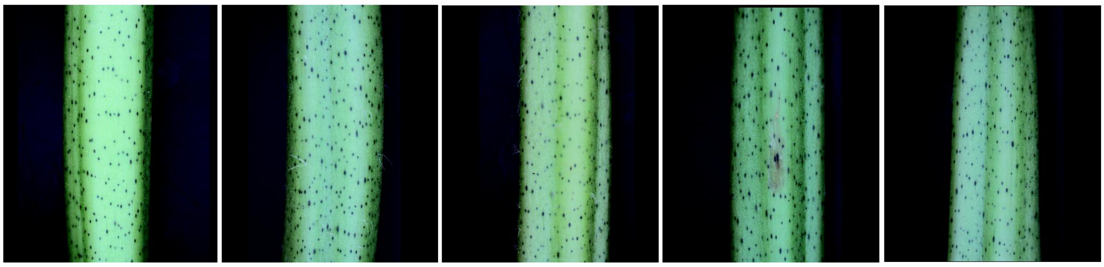

H7124xTM-1 H7124x9754-1 H7124xT Kuo H7124xNLD76 H7124xJS1

f

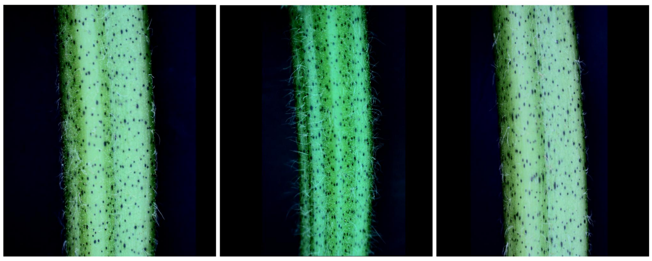

H7124x9741-3 H7124xXLZ53 H7124xNLD68

g

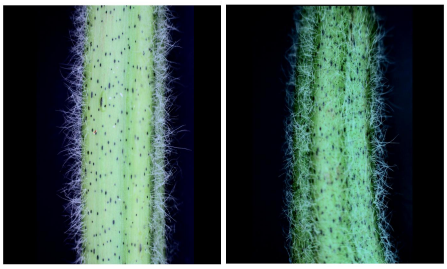

H7124xNLD56 H7124xT586

h

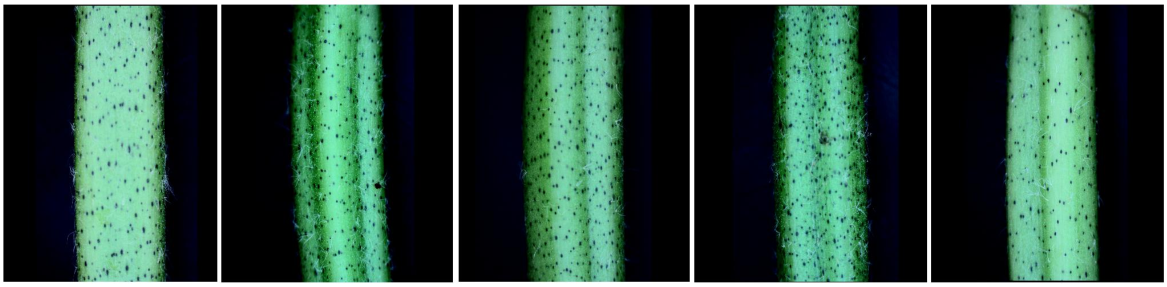

Aken4154xTM-1 Aken4154xTKuo Aken4154xJS1 Aken4154xNLD76 Aken4154x9754-1

i

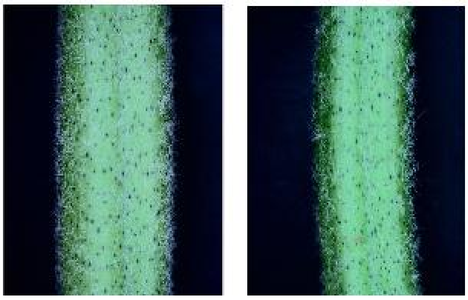

Aken4154xXLZ53 Aken4154xNLD68

j

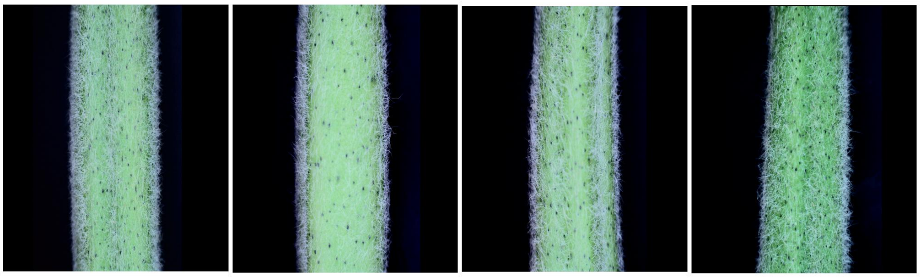

L-7009xTM-1 L-7009xT Kuo L-7009xNLD76 L-7009x9754-1

k

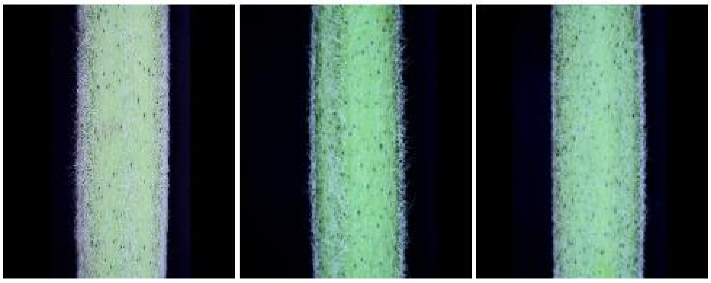

L-7009x9741-3 L-7009xXLZ53 L-7009xNLD68

l

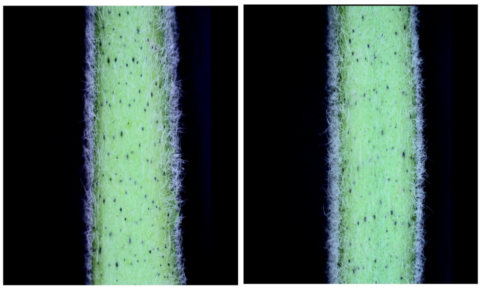

L-7009xNLD56 L-7009xT586

Fig. S5 Stem trichome phenotypes of parents and their F_1 hybrids from crosses between *G. hirsutum* and *G. babradense* varieties with different types of trichomes. **a.** Type I^a stems of four *Gh* parents; **b.** Type I^b stems of two *Gh* parents; **c.** Type II stems of two *Gh* parents; **d.** Type III and IV stems of two *Gb* parents; **e.** F_1 stems from crosses between H7124 (*Gb*, no trichomes) and type I^a *Gh* parents; **f.** F_1 stems from crosses between H7124 (*Gb*, no trichomes) and type I^b *Gh* parents; **g.** F_1 stems from crosess between H7124 (*Gb*, no trichomes) and type II *Gh* parents; **h.** F_1 stems from crosses between Aken 4154 (*Gb*, type III trichomes) and type I^a *Gh* parents; **i.** F_1 stems from crosses between Aken 4154 (*Gb*, type III trichomes) and type I^b *Gh* parents; **j.** F_1 stems from crosses between L-7009 (*Gb*, type IV trichomes) and type I^a *Gh* parents; **k.** F_1 stems from crosses between L-7009 (*Gb*, type IV trichomes) and type I^b *Gh* parents; **l.** F_1 stems from crosses between L-7009 (*Gb*, type IV trichomes) and type II *Gh* parents.
